# High resolution climate change observations and projections for the evaluation of heat-related extremes

**DOI:** 10.1038/s41597-024-03074-w

**Published:** 2024-03-01

**Authors:** Emily Williams, Chris Funk, Pete Peterson, Cascade Tuholske

**Affiliations:** 1 Climate Hazards Center, University of California, Santa Barbara, CA 93106 USA; 2 Sierra Nevada Research Institute, University of California, Merced, CA 95343 USA; 3 Department of Earth Sciences, Montana State University, Bozeman, MT 59717 USA; 4 Geospatial Core Facility, Montana State University, Bozeman, MT 59717 USA

**Keywords:** Projection and prediction, Natural hazards

## Abstract

The Climate Hazards Center Coupled Model Intercomparison Project Phase 6 climate projection dataset (CHC-CMIP6) was developed to support the analysis of climate-related hazards, including extreme humid heat and drought conditions, over the recent past and in the near-future. Global daily high resolution (0.05°) grids of the Climate Hazards InfraRed Temperature with Stations temperature product, the Climate Hazards InfraRed Precipitation with Stations precipitation product, and ERA5-derived relative humidity form the basis of the 1983–2016 historical record, from which daily Vapor Pressure Deficits (VPD) and maximum Wet Bulb Globe Temperatures (WBGT_max_) were derived. Large CMIP6 ensembles from the Shared Socioeconomic Pathway 2-4.5 and SSP 5-8.5 scenarios were then used to develop high resolution daily 2030 and 2050 ‘delta’ fields. These deltas were used to perturb the historical observations, thereby generating 0.05° 2030 and 2050 projections of daily precipitation, temperature, relative humidity, and derived VPD and WBGT_max_. Finally, monthly counts of frequency of extremes for each variable were derived for each time period.

## Background & Summary

Across the world, people are increasingly exposed to hydrometeorological and temperature extremes—including droughts and heat waves—with significant negative impacts on lives and livelihoods^1^. Anthropogenic climate change has increased the frequency and intensity of such hazards, exposing more people, places, and seasons to increasingly co-occurring hazards^2–4^. For instance, low precipitation in semi-arid areas combined with hot temperatures are leading to or exacerbating drought conditions^5,6^ while high temperatures coupled with humidity are exposing new populations to heat stress^7,8^. The projected increase in anthropogenic forcing for mid-century will likely increase the frequency, intensity, and spatio-temporal extent of such hazards. Given both the current and projected exposure of people and places to climatic hazards, particularly those with the lowest access to resources to support adaptation, the ability to estimate these variables grows increasingly important to support science-based adaptation and early warning efforts. Therefore, there is a growing need for high spatio-temporal resolution data on both current and projected hydroclimatic variables and extremes.

Using raw, coarse spatial resolution climate data is insufficient to resolve the unique and highly variable landscapes across which hazards occur. Furthermore, systematic biases in precipitation or temperature means can translate into large errors when driving impact models or calculating hazard indices. Relatively nearby places may have different baseline climatologies^9,10^, so an absolute change in a climatic variable (e.g. 2° Celsius of warming) may lead to or exacerbate hazards in one place but not the other. For example, in water-limited growing regions, crop yields will be sensitive to the amount of available soil moisture, which is tightly linked to the amount of growing season precipitation, which can vary across the landscape (e.g.^11–13^). Moreover, many crops are sensitive to very warm air temperatures^14–17^. Therefore, a localized two degrees of warming in a hot, dry place or season may have a much greater agro-pastoral impact than two degrees of warming in a cool, moist location^13,18^. Similarly, the changing magnitude of heat waves that impact human health and well-being can vary drastically over short distances^7^. In terms of human health and labor productivity, two degrees of warming in a hot, humid place may dramatically increase hazards and resulting impacts^19,20^, yet not increase hazards in a cooler or drier place. These dependencies can create strong, fine-grained links between geography, climate, and hazards. As coarse resolution data may not be able to distinguish climatic gradients across a landscape relevant to many human-environment systems, they are thus unable to capture more fine-scale variability in local climatic conditions. Therefore, high spatial resolution data that accurately captures extremes are crucial for monitoring such potential hazards.

However, in many areas of the world, there is a lack of high spatial resolution data. There is a very limited number of *in situ* weather observations for many of the most populated and least resourced countries^10^, which makes it difficult to monitor hydro-climatic extremes and their impacts on agriculture and human health. For example, there are only about 200 Climatic Research Unit (CRU) temperature observations across all of Africa^10^, and only about 800 available monthly rainfall stations (Fig. S1). This can be contrasted with the United States, where there are about 5,000 rainfall observations every month (Fig. S1). To some degree, these gaps can be filled with satellite-derived meteorological data, though many satellite-based data suffer from coarse resolution and inaccurately capturing conditions on-the-ground.

Several high-resolution temperature and precipitation products have been developed to address such global gaps. For example, the Climate Hazards InfraRed Precipitation with Stations (CHIRPS) precipitation data product^21^, and more recently, the Climate Hazards InfraRed Temperature with Stations (CHIRTS-daily) temperature data product^10,22^, were created to provide high resolution gridded estimates with global coverage. These two products rely on satellite inputs for two key components: high resolution (0.05°) long-term monthly average ‘climatologies’^9,10^, and high resolution (0.05°) time-varying satellite-only precipitation and temperature estimates. By using satellite data to develop high-resolution climatologies, these products have been shown to perform well with relatively low bias in data sparse regions like Colombia, Afghanistan, Ethiopia, and the Sahel compared to other products which create climatologies based on elevation and location^9^. Moreover, CHIRPS and CHIRTS-daily also benefit from consistent, long, moderate-resolution geostationary Thermal InfraRed (TIR) satellite observations, which hover over the equator at fixed longitudes and have been providing data since the early 1980s. These TIR values are combined with the CHC climatologies to produce good satellite-only precipitation^9^ and temperature estimates^10^. Therefore, these estimates are particularly valuable in data-sparse regions.

However, despite significant advancements in providing high-resolution temperature and precipitation data, there is still a lack of such global, high-resolution observational data for two variables relevant to human heat stress and drought: maximum wet bulb globe temperatures (WBGT_max_) and vapor pressure deficits (VPD), which are both related to temperature and RH. Warmer air can hold exponentially more water vapor^23,24^. Therefore, in places and seasons with ample available moisture, as temperature increases, evaporation will increase, maintaining or increasing RH^23^. This combination of high temperatures and humidity can lead to increased heat stress for people in hot, humid regions—measured here by WBGT. However, in water-limited environments, evaporation cannot keep pace, so a deficit will grow between the water holding *capacity* of the air and how much water vapor is actually held, producing greater evaporative demand—measured by VPD—and leading to or exacerbating drought.

WBGT is a widely-used heat stress metric^25,26^, which provides an estimate of ‘effective temperature’ for applications to human health in hot and humid areas^27^. WBGT measures what temperatures ‘feel like’ for a person exercising or doing work. As a function of wind speed, radiated heat, temperature, and humidity, it relates to how rapidly humans can cool their bodies through evaporation via sweat^27^. As a measure of heat stress, WBGT at the hottest time of the day—WBGT_max_—is particularly relevant. The advantage of using WBGT_max_, as opposed to meteorological indicators like T_max_, is that the former accounts for important nonlinear biophysical responses^28^. For humans, heat illness (hyperthermia) occurs when core body temperatures exceed 42 °C^29^. Improved sweating responses can help to lower core body temperatures yet are limited by high relative values^29,30^. Hot, humid air temperatures can limit the effectiveness of sweating to cool the body—30 °C WBGT_max_ has been associated with doubling in mortality rates compared to WBGT_max_ of 20 °C among vulnerable populations working outside^31^.

Conversely, in water-limited areas, decreasing RH—and therefore increasing VPD—can exacerbate drought conditions. VPD can directly exacerbate hydrologic drought by evaporating surface water bodies and decreasing soil moisture^32^. Moreover, it can limit plant productivity via two primary pathways: through the indirect effect of reducing soil moisture via increased evaporation, and through the direct effect of affecting stomatal conductance, or the rate of exchange of water vapor and carbon dioxide at the leaf-level^13^. Plants can sense environmental conditions and adjust their growth rate accordingly^13,18^. When soil moisture falls below critical thresholds, the plant will close stomata^14,33^. Moreover, if the plant senses increased VPD at the leaf-level, it will partially close stomata to conserve water. While these mechanisms allow plants to survive during dry times, it is at the expense of photosynthesis^34^, and can lead to lowered plant productivity, including for pasture and crops^13,16–18^.

The lack of available high-resolution data also exists for climate projections. There have been significant advancements in the accuracy and precision of climate models over the past two decades—climate models have reproduced observed temperatures for the past four decades^35^; newer versions of climate models are more accurate and precise than their predecessors^36^; and the scenarios underlying the projections are based on more refined models of human activity^37^. Yet, even with these advancements, relying on raw climate model output, alone, is insufficient for resolving highly-local hazards. Many climate models, such as those used by the Coupled Model Intercomparison Project (CMIP), have relatively coarse spatial resolution (typically ~100 km, see Table 1) and still contain biases^38^. Moreover, many models do not sufficiently account for internal climate variability which can lead to the under-identification of climate risk for certain sectors and places^39^.

**Table 1:** CMIP6 models and simulations.

Model	Institution	Spatial Resolution	Number of Simulations	
			SSP2-4.5	SSP5-8.5
ACCESS-CM2	CSIRO-ARCCSS	250 km	3	3
ACCESS-ESM1-5	CSIRO	250 km	11	10
CMCC-ESM2	CMCC	100 km	1	1
CNRM-CM6-1	CNRM-CERFACS	250 km	6	6
CNRM-CM6-1-HR	CNRM-CERFACS	50 km	1	1
CNRM-ESM2-1	CNRM-CERFACS	250 km	9	5
CanESM5	CCCma	500 km	25	25
CanESM5-CanOE	CCCma	500 km	3	3
EC-Earth3-CC	EC-Earth-Consortium	100 km	1	1
EC-Earth3-Veg	EC-Earth-Consortium	100 km	5	6
EC-Earth3-Veg-LR	EC-Earth-Consortium	100 km	3	3
FGOALS-g3	CAS	250 km	4	4
FIO-ESM-2-0	FIO-QLNM	100 km	3	3
GFDL-ESM4	NOAA-GFDL	100 km	3	1
GISS-E2-1-G	NASA-GISS	250 km	10	5
HadGEM3-GC31-LL	MOHC NERC	250 km	1	4
INM-CM4-8	INM	100 km	1	1
INM-CM5-0	INM	100 km	1	1
IPSL-CM6A-LR	IPSL	250 km	11	6
MIROC-ES2L	MIROC	500 km	30	10
MIROC6	MIROC	250 km	3	50
MPI-ESM1-2-HR	MPI-M DWD DKRZ	100 km	2	2
MPI-ESM1-2-LR	MPI-M AWI DKRZ DWD	250 km	9	10
MRI-ESM2-0	MRI	100 km	1	1
UKESM1-0-LL	MOHC NERC NIMS-KMA NIWA	250 km	5	5

Table 1 lists the model name, source institution number of simulations, and spatial resolution of the simulations used in creating the CHC-CMIP6 dataset.

In response to the challenges presented by limited available data on climatic hazards in data-sparse regions, the Climate Hazards Center Coupled Model Intercomparison Project Phase 6 climate projection dataset (CHC-CMIP6) was created to provide high-resolution observation and scenario-based projection hydro-climatic data that can be used to support the identification, monitoring, and analysis of current and future potential hazards in data sparse regions. The dataset retains the high spatial resolution and precision of the CHC temperature and precipitation products while projecting changes in hydro-climatic variables using climate model output. The CHC-CMIP6 dataset offers high-resolution (0.05°), global daily hydro-climatic data—minimum temperature (T_min_), maximum temperature (T_max_), precipitation, relative humidity (RH), VPD, and WBGT_max_—for the current/observed period (1983–2016) and near-future scenarios (2030 and 2050). It also provides counts of T_max_, T_min_, precipitation, VPD, and WBGT_max_ ‘extremes’ (Section 2.4) for each of the scenarios.

**Fig. 1:**
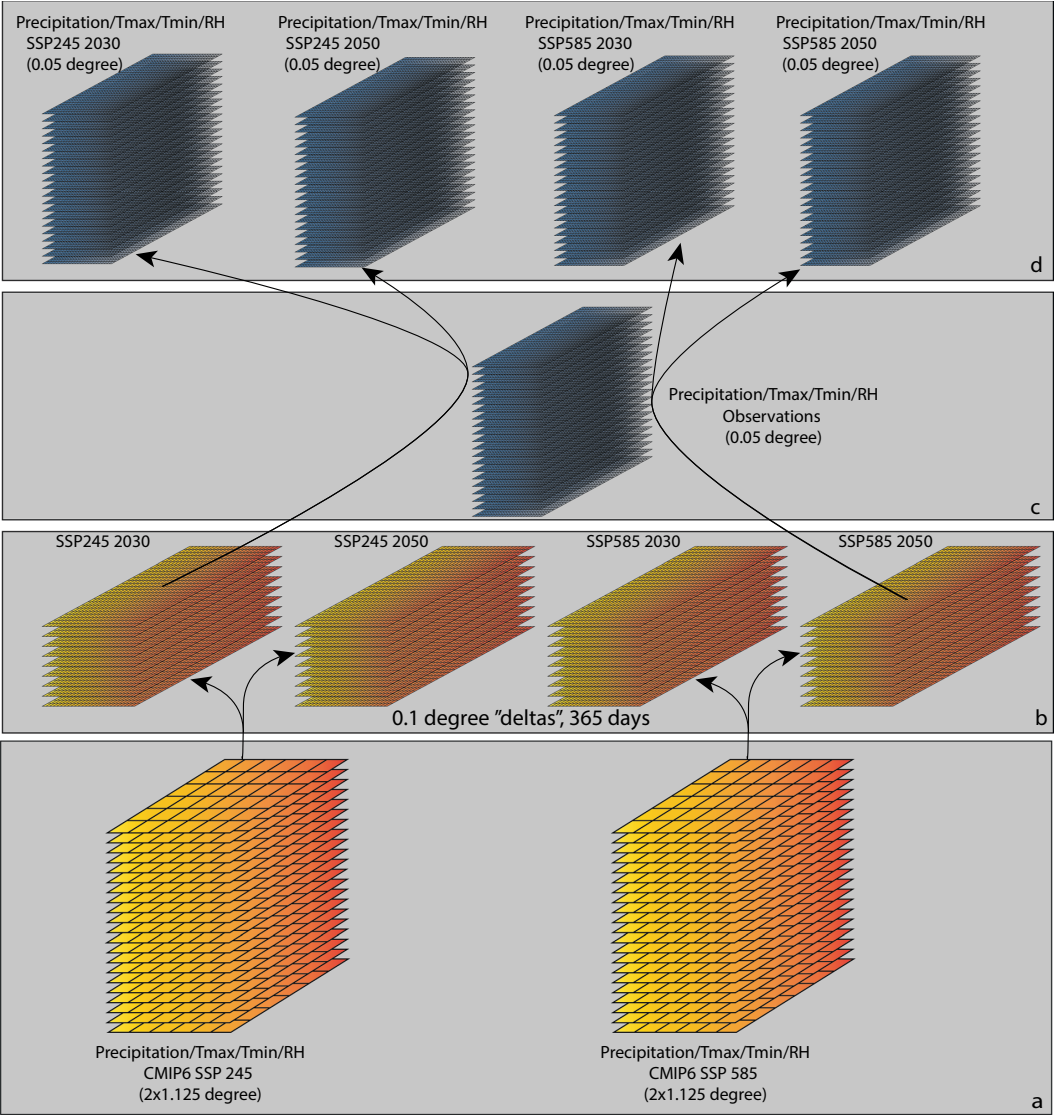
Process schema for the development of the CHC-CMIP6 0.05° datasets. Large ensembles of CMIP6 precipitation, T_max_, T_min_, and RH simulations (**a**) were used to calculate monthly “delta” (change) estimates for 2030 and 2050, which are reprojected in space and time to produce global 0.1° daily delta files (**b**). The daily deltas were then combined with observed precipitation, T_max_, T_min_ and RH (**c**) to produce high resolution (0.05°), time-varying data sets incorporating the CMIP6 changes between 1983–2016 and 2025–2035 and 2045–2055 (**d**).

The CHC-CMIP6 dataset builds on high-resolution temperature and precipitation products developed by the University of California, Santa Barbara Climate Hazards Center; RH data derived from ERA5; and large ensembles of coarse resolution CMIP6 simulations of temperature, precipitation, and RH^40^. As depicted in Fig. 1, these data were used to create high-resolution projections using the following process: The CMIP6 multi-model ensemble simulations (Fig. 1a) were used to calculate 2030 and 2050 ‘deltas’—or mean changes between the observational and projection periods—for temperature, precipitation, and RH (Fig. 1b). Next, these ‘delta’ files were combined with the high-resolution observations (Fig. 1c) to produce high-resolution 2030 and 2050 projections of temperature, precipitation, and RH for the two CMIP6 scenarios (SSP2–4.5 and SSP5–8.6) (Fig. 1d). WBGT_max_ and VPD were then derived for the observational (1983–2016) period using the observed temperature and RH fields, and for the projections using the projected temperature and RH fields. Finally, data layers depicting counts of extremes were calculated for the observational period and the four projections.

## Methods

### Underlying data products

#### The CHC precipitation (CHIRPS) and temperature (CHIRTS) products

The CHC-CMIP6 dataset is partially based on the Climate Hazards Center (CHC) precipitation and temperature products: the Climate Hazards InfraRed Precipitation with Stations (CHIRPS) product^21^ and the Climate Hazards InfraRed Temperature with Stations (CHIRTS) product^10,22^. The CHC has developed a three-step process that leverages the power of satellite meteorological observations and information from *in-situ* weather stations to create the high-resolution CHIRPS and CHIRTS products. The use of satellite observations allows for creating high resolution data which captures valuable information about local changes in meteorological variables, especially in data sparse regions of the global south, which is where many of the impacts of climate change are likely to be, and have been, felt most keenly. For example, in Ethiopia, where mean precipitation changes dramatically over short spatial distances, the local variations in climate conditions are well-captured by CHIRPS and its underlying climatology^9^.

The first step in this process involves the development of high resolution (0.05°) precipitation and temperature climatologies^9^. The Climate Hazards Center precipitation climatology (CHP_clim_) was derived by combining satellite-based rainfall estimates with station data and elevation. The CHIRTS climatology product (CHT_clim_) is similarly based on cloud-screened satellite observations of thermal infrared brightness temperatures, which also provides detailed information related to meteorological conditions on the ground, even in areas with sparse observation networks^10,22^. As spatial variogram analysis has shown^10^, the density of monthly air temperature observations in many parts of the world are far from sufficient to support the mapping of monthly air temperature anomalies. Geostationary satellites, however, provide long, rapidly repeated thermal infrared observations of the Earth’s surface at fairly high spatial resolutions. Hence, the next step involves combining gridded 0.05° geostationary thermal infrared data (TIR) with the 0.05° climatologies to produce satellite-only CHIRT and CHIRP. Ultimately, CHIRP relies on the satellites’ ability to see cold cloud tops, while CHIRT relies on direct observations of the non-cloudy land surface. Both types of observations take advantage of the fact that cloud tops tend to be much colder than surface temperatures. The final step involves the addition of station observations, resulting in the CHIRPS and CHIRTS. The development process underlying these products, however, is different. It is relatively easy to obtain reliable and rapidly updated satellite-observations of cloud top temperatures, supporting rapid updates of the CHIRPS. Obtaining reliable estimates of surface temperatures is more difficult and requires inter-satellite calibration. The temperatures of cold cloud tops, used to calculate CHIRPS, are very cold, and a small bias between satellites, or a small change in bias due to sensor or orbit degradation, produces only minor changes in precipitation estimates. Surface temperatures, on the other hand, have variations that can be strongly influenced by relatively small biases. The correction of these biases requires careful satellite-specific inter-calibrations^10^.

CHIRPS and CHIRTS have different temporal coverages and should be analyzed at different temporal resolutions. While CHIRPS is updated to near-present, the daily CHIRTS product currently ends in 2016, with near-term plans to update both CHIRTS-daily and the CHC-CMIP6 dataset through near-present. Moreover, CHIRTS supports the analysis of daily or monthly temperature. However, CHIRPS should be analyzed at the monthly timescale as this is the accumulation period for which the product is supported by *in situ* rain gauge observations^9,41^. These considerations carry over into the CHC-CMIP6 dataset.

#### ERA5-derived relative humidity

The CHC-CMIP6 dataset also includes RH derived using hourly air temperature (Ta) and dewpoint temperature (Td) from the ERA5 reanalysis^42^ and daily minimum and maximum CHIRTS (CHIRTS-daily_Tmin_, CHIRTS-daily_Tmax_). RH was calculated as the ratio of actual vapor pressure (AVP)—a function of the actual water content of the air—to saturation vapor pressure (SVP)—or the water holding *capacity* of the air at a certain temperature^43^, in equations^1–3^. SVP is an exponential function of air temperature, while AVP is an exponential function of dewpoint temperature. This dewpoint temperature-based approach to calculating RH was selected due to its straightforward computational method and to allow direct comparisons *in situ* station observations for the validation of the dataset, as described in Section 3.2.

1 $$SVP={0.6111}\ast {\exp }{(}{17.3}\ast Ta/{(}Ta+{237.3}{)}{)}$$

2 $$AVP={0.6111}\ast {\exp }{(}{17.3}\ast Td/{(}Td+{237.3}{)}{)}$$

3 $$RH={100}\ast AVP/SVP$$

Two values of RH were calculated: RH at the hottest hour of the day (RH_x_) for calculating WBGT_max_ and daily average RH (RH_ave_) for calculating VPD. To derive both RH values, first, the hourly ERA5 fields were resampled to 0.05 degrees using bilinear interpolation to the same resolution as the CHIRTS products. For RH_x_, ERA5 Td was selected for each day at the hour of maximum ERA5 Ta (Td_Tmax_). RH_x_ was then calculated using Td_Tmax_ and CHIRTS_Tmax_. For RH_ave_, the daily mean ERA5 Td was calculated and used with the average of CHIRTS_Tmin_ and CHIRTS_Tmax_. For both calculations, for cases where Td was greater than the corresponding temperature field (CHIRTS_Tmax_ or CHIRTS_Tave_) (RH > 100%), Td was set to equal that temperature field (RH = 100%). Notably, we calculate mean daily SVP and AVP from mean daily Ta and Td, respectively, instead of hourly SVP and AVP as a function of hourly Ta and Td, in order to make use of our CHIRTS product. As AVP and SVP are nonlinear equations, using daily means presents a potential source of error, though the error is ~ +/− 1 percentage point.

Note that while minimum RH will often occur at the hour of maximum temperatures, this is not always the case. In the ERA5 data, there are numerous instances in which the hour of maximum Ta is different from the hour of minimum Td, or the difference between Ta and Td varies drastically throughout the day. For these locations, RH_ave_ may thus be lower than RH_x_. Moreover, the computed daily RH_x_ fields have visible artifacts. These arise due to the fact that the hour of RH_x_ may differ significantly from pixel to pixel. For the purposes of calculating WBGT_max_, these values of RH_x_ are acceptable. Nonetheless, we detail possible uncertainty in WBGT_max_ that may arise from errors in our RH_x_ estimation (Supplementary Text S2, Figs. S2, S3).

#### The coupled model intercomparison project phase 6 (CMIP6) model output

As the magnitude of future increases in radiative forcing is dependent on both the current and future rate of emissions, and is therefore not fixed, we use two scenarios from CMIP6—Shared Socioeconomic Pathway (SSP) 2–4.5 and 5–8.5^37^. The SSP245 scenario is based on ‘middle-of-the-road’ projections of development (SSP2), resulting in 4.5 W m^−2^ of radiative forcing by 2100. The SSP585 scenario projects rapid fossil fuel development and increased global market integration (SSP5), which is expected to result in 8.5 W m^−2^ of radiative forcing by 2100. These are generally considered the most-likely scenario (SSP245) and the high-emissions scenario (SSP585)^44,45^.

CMIP6 multi-model ensemble simulations were accessed from Lawrence Livermore National Laboratory (LLNL) node of the Earth System Grid Federation (ESGF) platform (https://esgf-node.llnl.gov/search/cmip6/). Monthly average near-surface relative humidity (hurs), near-surface minimum and maximum temperature (tasmin, tasmax), and precipitation (pr) were retrieved for 25 models across three experiments—historical (1850–2014), and SSP245 and SSP585 (2015–2100). The average equilibrium climate sensitivity (ECS) of the 25 models used in this study (~3.86) falls within the most likely range of ECS (2.2–4.9 °C, 90% chance)^46^. Within each model, the same ensemble members were selected for the historical and future scenario experiments. In total, 152 simulations across 25 models were retrieved for the historical and SSP245 experiments, while 167 were retrieved across the same 25 models for the historical and SSP585 (Table 1).

Next, spatio-temporal cubes (1850–2050) were created for each variable (hurs, tasmax, tasmin, pr) and experiment (SSP245 and SSP585) (Fig. 1a). Historical and projection data were stacked using the netCDF Record Concatenator utility (ncrcat). The cubes were then resampled to 100 km resolution using bilinear interpolation with the Climate Data Operator (CDO) software *remapbil* utility. To avoid over-weighting any individual model, for each variable and scenario, the mean was taken for each model across all simulations to derive the model mean, and then the multi-model mean was taken to derive the ensemble mean^47^. As such, four projection scenarios were created—2030_SSP245, 2030_SSP585, 2050_SSP245, and 2050_SSP585.

### Deriving projections

Projected data were created by perturbing the CHC observational data with ‘delta’ (i.e. change) fields derived from the CMIP6 simulations. This approach therefore retains the precision and variability present in the observational data. It moreover limits the effect of any biases in the CMIP6 temperature fields by focusing on simulation- and model-specific changes rather than raw values: the projected warming trend in CMIP6 models has been found to be correlated with modeled observed warming trends^38^, meaning any potential bias in CMIP6 would stay relatively constant over time.

#### Delta fields

Daily ‘deltas’ for T_min_, T_max_, RH_ave_, and precipitation were derived for each grid cell, representing the change in each variable between the observational and projection periods for the four CMIP6 scenarios (2030_SSP245, 2030_SSP585, 2050_SSP245, 2050_SSP585) (Fig. 1b). First, monthly ‘deltas’ were calculated from the CMIP6 spatio-temporal cubes as the difference between the monthly means of the observational period (1983–2016) and the monthly means of the projection periods (2025–2035, 2045–2055). The T_max_, T_min_, and RH_ave_ deltas were calculated as monthly arithmetic differences (Eq. 4), while the precipitation deltas were calculated as ratios (Eq. 5). Ratios were used for precipitation because this field is heteroscedastic and bounded at zero (i.e. precipitation cannot be negative).

4 $$\begin{array}{c}\Delta 203{0}_{{\rm{monthly}}}={\rm{Mean}}{(2025-2035)}_{{\rm{monthly}}}-{\rm{Mean}}{(1983-2016)}_{{\rm{monthly}}}\\ \Delta 205{0}_{{\rm{monthly}}}={\rm{Mean}}{(2045-2055)}_{{\rm{monthly}}}-{\rm{Mean}}{(1983-2016)}_{{\rm{monthly}}}\end{array}$$

5 $$\begin{array}{c}\Delta 203{0}_{{\rm{monthly}}}=[{\rm{Mean}}{(2025-2035)}_{{\rm{monthly}}}+\varepsilon ]/[{\rm{Mean}}{(1983-2016)}_{{\rm{monthly}}}+\varepsilon ]\\ \Delta 205{0}_{{\rm{monthly}}}=[{\rm{Mean}}{(2045-2055)}_{{\rm{monthly}}}+\varepsilon ]/[{\rm{Mean}}{(1983-2016)}_{{\rm{monthly}}}+\varepsilon ]\end{array}$$

*Precipitation is in units of millimeters per month, and ε is set to 7 millimeters, a value found reasonable based on the production of the CHIRPS dataset*.

For each pixel, variable, and climate change scenario (∆2030_SSP245, ∆2030_SSP585, ∆2050_SSP245, ∆2050_SSP585), the monthly deltas produced from Eqs. 4 and 5 were temporally downscaled to daily delta values using the following process. For a 14-month period—including the preceding and following month of the year (Dec_year-1_ … Jan_year+1_)—the monthly delta value was first assigned to each of the daily values for that month. This 14-month-long daily time series was iteratively smoothed 10 times with a seven element moving average (boxcar) filter to obtain a smooth seasonal progression. Then, the first and last month were discarded, resulting in 365 daily delta values for each location, variable and scenario. These daily data were then further adjusted so that the daily data mean (temperature and RH) or sum (precipitation) across each month equals the original monthly value, thus resulting in a time series which realistically represents the observed data. These daily deltas were rescaled from 100 km to a global 0.1° resolution. This procedure was selected because it provides a non-parametric means of generating a linear, smoothly varying de-convolution of the original 12 monthly delta values.

#### Creating the projections

The daily observational T_min_, T_max_, RH_x_, RH_ave_, and precipitation data were perturbed using the corresponding daily delta fields to create the projected data. A single, stationary delta value was used for each day, location, and variable. For example, the same January 1^st^ T_max_ delta was applied to the 1983 January 1^st^ T_max_ as the 2016 January 1^st^ T_max_ value. Therefore, these datasets correspond to the same period of record as the original observations. For air temperature and RH, the delta fields were added to the observational fields, resulting in 34 years of daily projections for 2030 (2030_SSP245, 2030_SSP585) and 2050 (2050_SSP245, 2050_SSP585). Note that the RH_ave_ deltas were used to perturb both the observational RH_x_ and RH_ave_ fields. For precipitation, as the deltas were calculated as ratios, the observational fields were multiplied by these deltas. Moreover, for precipitation, only monthly data are provided and analyzed. Because it relies on an indirect proxy for precipitation (TIR), which cannot sense hydrometeors (unlike microwave satellites), CHIRPS is not well-suited to the analysis of sub-monthly precipitation extremes (41; see section 2.1.1).

No attempt was made to detrend the underlying observational datasets. The benefit of the chosen approach is transparency: we can explicitly map and understand the delta fields, and their implications for changes in the distribution of the daily temperature or monthly precipitation values.

### Deriving VPD and WBGT observations & projections

Both WBGT_max_ and VPD were calculated for the observational and projection scenarios using the temperature and relative humidity fields from the respective scenarios. We calculated WBGT_max_ because we are interested in peak heat stress values that typically occur in the afternoon when air temperatures and relative humidity typically reach their warmest and lowest values, respectively. WBGT_max_ therefore is constructed with daily T_max_ and RH_x_^48^. Following previous work^7^, we first calculated Heat Index (HI_max_) values with T_max_ and RH_x_ according to National Ocean and Atmospheric Administration’s (NOAA) guidelines^49^. Equations for HI_max_ may be found in the NOAA^49^ reference and in the accompanying code for this manuscript. The HI_max_ values were used to estimate WBGT_max_^50^. The full WBGT estimation process includes radiant heat and wind speeds^25^, but indoor or shaded WBGT (°C) with fixed air speeds (0.5 m s^−1^) can be estimated closely by a quadratic transform of daily HI_max_ (°F) values (^50^; Eq. 6).

6 $$WBG{T}_{{max}}(^\circ {\rm{C}})=-0.0034\ast H{I}_{{max}}^{2}(^\circ {\rm{F}})+0.96\ast H{I}_{{max}}(^\circ {\rm{F}})-34$$

While our objective was to produce WBGT_max_, we also provide the HI_max_ observational record and HI_max_ projections in the CHC-CMIP6 dataset. The observed (1983–2016) WBGT_max_ and HI_max_ fields were calculated using the above equation and the observed T_min_, T_max_, and RH_x_ fields, while the projected WBGT_max_ and HI_max_ fields were derived from the four scenarios of T_min_, T_max_, and RH_x_. We note that the WBGT_max_ observations differ from previous work^7^ because we updated the parameterization of RH_x_ used here (Section 2.1.2; Supplementary Text S1). Furthermore, as noted above, we overview the range of possible uncertainty in HI_max_ and WBGT_max_ that may arise from errors in our RH_x_ estimation for extreme hot-dry and hot-humid conditions relevant to heat impacts to human health and well-being (Figs. S2, S3).

We are interested in average daily VPD, however, as it is the prolonged effect of high VPD throughout the day which can lead to depleted soil moisture, stomatal closure, and overall reduced productivity or increased plant stress. VPD therefore was calculated using daily T_ave_ and RH_ave_. VPD is the arithmetic difference between SVP and AVP^43,51^ (Eq. 7). To derive VPD, first SVP_ave_ was calculated by taking the mean of SVP_min_ and SVP_max_ (which were derived using Eq. 1), while AVP_ave_ was derived from RH_ave_ and SVP_ave_ (Eq. 8). The observed VPD fields were calculated using observed T_min_, T_max_, and RH_ave_, while the projected VPD fields used the projected T_min_, T_max_, and (RH_ave_).

7 $$VPD=SVP-AVP$$

8 $$AVP=RH\ast SVP/100$$

### Deriving extreme counts

Counts of the number of extreme days per month were calculated for T_min_, T_max_, WBGT_max_, and VPD for the observations and four scenarios (2030_SSP245, 2030_SSP585, 2050_SSP245, and 2050_SSP585). For these fields, extreme counts are based on daily data, processed on a monthly basis. Validations of daily T_min_ and T_max_ have documented strong performance^22^, and as shown later in Sect. 3.2, we identified similar strong performance for VPD and WBGT_max_. For these variables, data are available by month, depicting the number of days for each month experiencing an ‘extreme’.

Precipitation extreme counts are calculated on a monthly basis for two reasons: (1) defined extreme thresholds exist on a monthly, not daily, timescale (see below), and (2) confidence in the CHIRPS product is much higher at the monthly scale than the daily scale, as daily CHIRPS has been shown to underestimate precipitation extremes^41^. For precipitation, the data layers depict whether the total monthly value for that pixel is classified as an extreme. The focus on monthly extremes aligns with the design focus of the CHIRPS product—seasonal crop and rangeland drought stress.

Definitions of extremes for each variable were based on two methods: based on known thresholds (Sect. 2.4.1) and by calculating pixel-specific breakpoints using the 95^th^ and 99^th^ percentile (Sect. 2.4.2).

#### Calculating threshold-based extremes

A series of fixed thresholds for each variable were identified for temperature, precipitation, WBGT_max_. and VPD. Two thresholds were used for T_min_ and T_max_: 30 °C and 40.6 °C, respectively, representing moderate and extreme heat exposure. These were chosen based on documented thresholds for agricultural and human heat stress. 30 °C has been used to identify agricultural heat stress as inhibition of photosynthetic activity generally begins at or above 30 °C^52^. In health research, heat stroke is defined by when human core body temperature reaches or exceeds 40.6 °C^53^. This threshold has been used in studies identifying extreme heat events due to its relationship to human physiology^54^. For precipitation, the threshold of 100 mm per month was utilized. This threshold was selected based on typical monthly crop water requirements used in crop models^55^.

Two thresholds were used for WBGT_max_: 28 °C and 30 °C^7^. A WBGT_max_ value of 28 °C is defined by the International Standards Organization as the threshold for risk of heat-related illness for acclimated persons at moderate metabolic rates, while 30 °C is the same threshold for a group of persons at low metabolic rates^56^. Finally, we defined three thresholds for VPD: 2 kPa, 3 kPa, and 4 kPa. Studies have found that stomatal closure begins when VPD increases above 2 kPa^16,57,58^, and gross primary productivity exhibits a decline for sites and times when VPD exceeds 2 kPa^58^. When VPD surpasses 3 kPa, evaporative demand can be too high for certain plant types to offset via mitigation strategies^59^. Furthermore, few sites experience VPD greater than 4 kPa.

For the temperature-related variables (T_min_, T_max_, VPD, and WBGT_max_), for each variable, year, and scenario, the number of days surpassing each variables’ thresholds were calculated. For precipitation, the number of months below the fixed threshold were calculated. In other words, the precipitation extremes data will be coded as either ‘1’ (extreme) or ‘0’ (not extreme), which may be analyzed over time by adding these extremes over seasons, years, or other time steps. For the observations and the four CMIP6 scenarios, extreme counts and means are provided for each month, for each year. For the convenience of the users, annual and 1983–2016 count totals are also provided.

#### Calculating percentile-based extremes

Extremes were also calculated using percentiles. Monthly percentiles were calculated for each pixel based on the observational data, using a 1981–2010 baseline period. For T_min_, T_max_, VPD, and WBGT_max_ for each pixel, the daily 95^th^ and 99^th^ percentiles were calculated using 1983–2016 daily data, resulting in a 95^th^ and 99^th^ percentile value for each variable. For each of these four variables, each year (1983–2016), and each of the five scenarios, the number of days for each month were calculated at each pixel that surpass these percentile-defined extreme values. Users should be aware that the number of observations used to describe the warmest 1% of the datasets is small (10 or 11 observations), hence the uncertainty associated with precise 99th percentile breakpoints is substantial^60^. However, as described in the usage notes, despite this uncertainty the data can still provide valuable information about the spatial and temporal evolution of risk. For precipitation, both low and high percentile-based extremes were calculated: the corresponding values for monthly precipitation falling lower than the 20^th^ percentile and higher than the 90^th^ percentile were calculated for each month. Caution should be taken to only utilize counts of low precipitation extremes for wet places and seasons, and to not over-analyze percentile-based precipitation extremes in dry seasons or months: in a dry month or season, an extreme percentile value may be associated with a very small anomaly, in terms of an absolute value in mm.

## Data Records

The CHC-CMIP6 dataset is available for free use via ftp at 10.21424/R47H0M^61^. The dataset is under a Creative Commons Attribution 4.0 International (CC BY 4.0) license.

The data is available as GeoTIFF files. Each file is 25MB, and contains data for a single month, variable, and scenario at 0.05° resolution. The naming convention on files is SCENARIO.VAR.YYYY.MM.DD.tif (e.g. the file “2030_SSP245.RH.1983.01.01.tif” is the RHave data for Jan 1 1983 for the 2030 SSP 245 scenario). The data files are organized first by scenario (observations, the four projection scenarios, and extremes), then by variable, and finally by year. Further description of each of the files is included in the accompanying README file. To facilitate analysis, annual and long-term-average subdirectories in the Tmin, Tmax, VPD, and WBGTmax folders also contain annual and long-term average (34 year) counts for each of the extreme categories and the monthly mean fields.

## Technical Validation

To validate the CHC-CMIP6 data layers, we examined accuracy in two stages. First, we tested for accuracy in the observational CHC data layers—this is not only important for the observational data, but also for the projections as the projection data layers are based on perturbations of the observational data. This included an evaluation of bias as well as temporal fidelity (correlation) and Mean Absolute Errors (MAE). Second, we compared the CHC-CMIIP6 biases with the raw CMIP6 data. When and where the CHC-CMIP6 fields reduce bias, they may give more reliable projections than the CMIP6 fields.

This validation focuses on daily T_min_, T_max_, RH, and the derived VPD and WBGT_max_. While some evaluation of the daily T_max_ values has been previously provided^7,22^, we examine here for the first time RH and the derived variables at daily time steps. This focus on temperature-related variables was selected because we expect the improved analyses of variables to be the key feature of this CHC-CMIP6 dataset. Evaluation of the monthly observational precipitation data (CHIRPS) can be found in other manuscripts^9,21^.

### Validation data

Validation of the observational data layers was based on using station observations from the Global Summary of the Day (GSOD) provided by NOAA’s Climate Prediction Center. Daily minimum and maximum temperatures (T_min_, T_max_) and dewpoint temperature (T_d_) were retrieved from GSOD for 1983–2016, for a total of 15,908 stations (T_min_ = 15,907, T_max_ = 15,908, T_d_ = 9,582). A station screening process was applied to GSOD data, following previous work^22^.

1. All stations with fewer than 2,920 observations (8 years of data) were discarded, to ensure long enough temporal coverage in the GSOD validation data.
2. All stations that had a high rate of reporting of exactly 0 were discarded. Stations may record 0 if there is no data. To minimize the likelihood of false zero contamination in the validation data, if stations reported exactly 0 more than 15% of the time, or the average across all months or years was exactly 0, the station was discarded.
3. The T_max_ GSOD data were compared to Climate Hazards Center T_max_ climatology (CHT_clim_). All GSOD stations were discarded for which the corresponding CHT_clim_ value was NA. Furthermore, as there can be issues within the GSOD dataset–with spatial location being one very common issue, especially in areas with complex topography–GSOD station data were screened for instances where the GSOD and CHT_clim_ T_max_ were substantially different, defined by satisfying either of the below conditions (which removed 684 stations):
  - abs(m - CHT_clim_) > 5 °C *(m = station median)*
  - abs(m - CHT_clim_)/s > 3 (*m* = *station median; s* = *station standard deviation*)
4. Finally, as T_d_ should not fall above air temperature, all values of T_d_ greater than T_ave_ were set to the value of T_ave_.

This screening process resulted in a remaining 9,755 T_min_, 8,529 T_max_, and 7,498 T_d_ stations. Of the GSOD stations remaining after the screening process, only the sub-set of stations that were retained across T_min_, T_max_, and T_d_ were used in the validation, a total of 6,649 stations.

For the 6,649 GSOD stations, the GSOD T_min_, T_max_, and T_d_ observations were subset to the local hottest 3-month period, defined using CHT_clim_. Finally, these resulting observations for the three variables were screened, by removing daily observations which deviated dramatically from the climatological average of the GSOD data. To do so, z-scores were calculated for each observation, and observations were retained only if they satisfied the following conditions:

-4 < z < 4.5
- x - m < 20 °C (*m* = *station monthly median; x* = *station observation*)

### Technical validation of observational fields in CHC-CMIP6 dataset

To validate the CHC-CMIP6 observational fields, the data were compared with GSOD station data and the raw CMIP6 fields for the local hottest 3-month period for 1983–2016. The CHC-CMIP6 fields for all five variables (T_min_, T_max_, RH_ave_, VPD, and WBGT_max_), and raw CMIP6 T_min_, T_max_, and RH_ave_ fields, were extracted for the location of each GSOD validation station using bilinear interpolation of the four nearest pixels. Note, the observational CHC-CMIP6 temperature fields are from the CHIRTS products, so the validation statistics for those fields are recalculated here, yet align with earlier validation efforts^22^.

We first tested the observational data for accuracy by comparing the daily observational CHC-CMIP6 fields and those from GSOD station data. Corresponding variables were derived where necessary. GSOD RH_ave_ was derived from GSOD T_min_, T_max_, and T_d_. VPD and WBGT were calculated for both GSOD and CHC-CMIP6 using the corresponding T_min_, T_max_, and RH_ave_ fields. Note, average WBGT, instead of WBGT_max_, was derived as only average dewpoint temperature was available from GSOD. The data were then converted to monthly anomalies by subtracting the mean monthly climatological value from each corresponding daily value. For the location of each GSOD station, Spearman’s correlation coefficients and associated p-values, and mean absolute error (MAE) were calculated for the five variables between the two datasets.

**Fig. 2:**
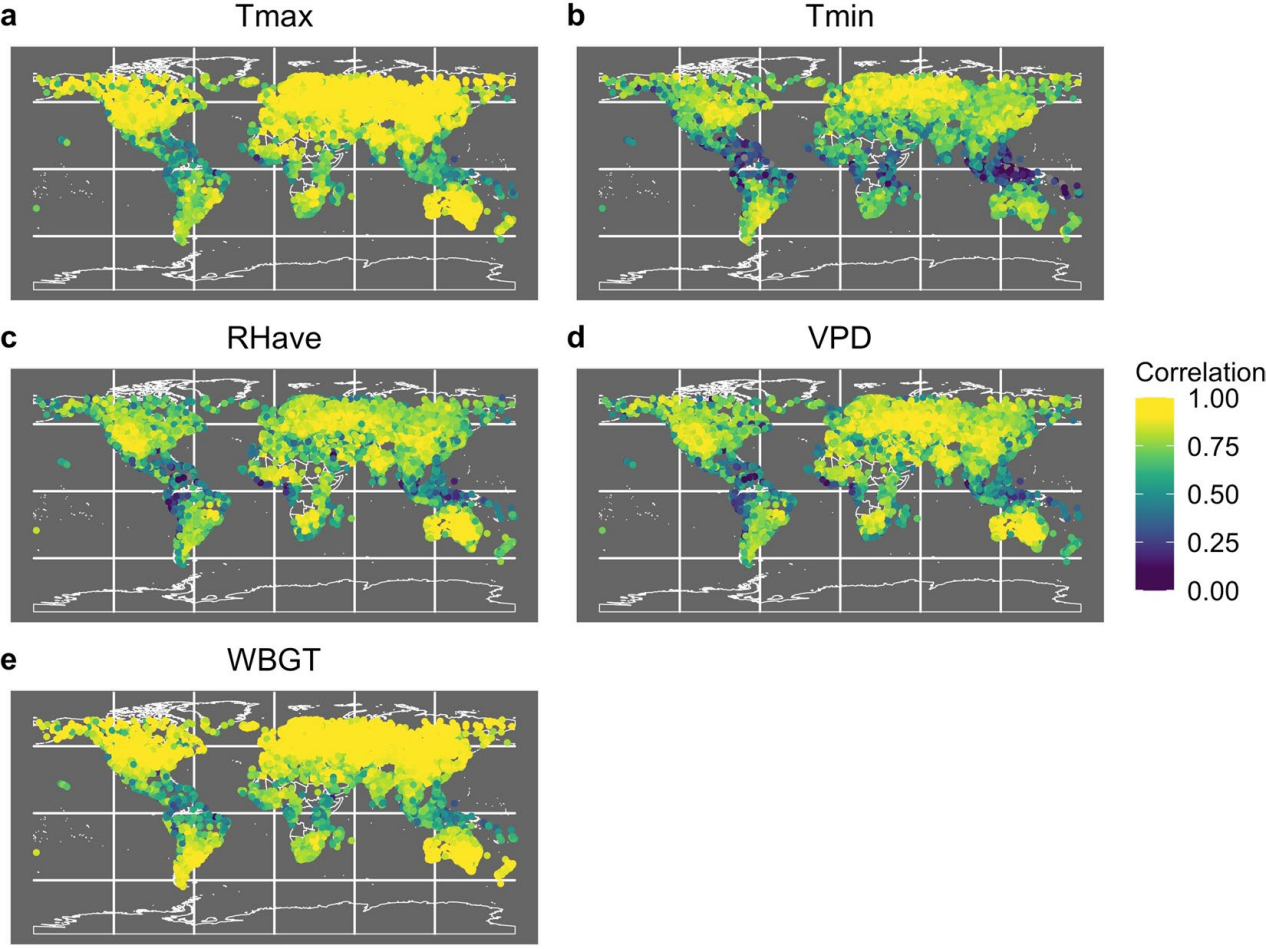
Map of correlation between CHC-CMIP6 and GSOD for each variable. Each point depicts the correlation coefficient between CHC-CMIP6 observational fields and GSOD at the location of each GSOD station.

Table 2 and Fig. 2 present the validation statistics, broken up by continent and shown as a global average. The CHC-CMIP6 observational data perform well for each of the five variables globally as well as regionally. In particular, T_max_ and WBGT have a particularly high correlation with GSOD (0.86 globally). The underlying CHIRTS_min_ is slightly warmer than GSOD T_min_ while the CHC-CMIP6 observational RH_ave_ is slightly lower than GSOD RH_ave_ (Table 3), which in turn influences VPD, yet there is still high agreement for the three variables, with global average correlations at 0.73–0.76.

**Table 2:** Validation Statistics.

	T_max_		T_min_		RH_ave_		VPD		WBGT	
	Cor	MAE	Cor	MAE	Cor	MAE	Cor	MAE	Cor	MAE
Global	0.86	1.1	0.73	1.3	0.76	4.83	0.76	0.15	0.86	0.77
Africa	0.80	1.07	0.67	1.28	0.75	4.78	0.76	0.19	0.78	0.72
Asia	0.89	0.95	0.72	1.15	0.75	4.44	0.77	0.15	0.86	0.71
Europe	0.95	0.96	0.84	1.24	0.8	4.89	0.82	0.11	0.95	0.7
North America	0.86	1.28	0.75	1.49	0.76	5.14	0.73	0.14	0.89	0.87
Oceania	0.92	1.00	0.73	1.47	0.83	4.51	0.8	0.15	0.91	0.79
South America	0.74	1.33	0.66	1.16	0.69	5.18	0.69	0.17	0.79	0.84

Table 2 depicts the median Spearman’s correlation coefficient (Cor) and the mean absolute error (MAE) for daily data by continent during the hottest 3-month period. All correlations are significant (p-value < 0.01). The CHC-CMIP6 observational products perform well for each of the five variables globally as well as regionally. The spatial distribution of correlations may be seen in the SI.

**Table 3:** Mean climatological differences.

	T_max_		T_min_		RH_ave_	
	GSOD - CHC-CMIP6	GSOD - raw CMIP6	GSOD - CHC-CMIP6	GSOD - raw CMIP6	GSOD - CHC-CMIP6	GSOD - raw CMIP6
Global	−0.08	1.45	−2.21	−0.91	4.43	−0.68
Africa	−0.13	1.12	−2.23	−0.36	3.71	0.8
Asia	−0.16	1.98	−1.86	0.1	4.62	−0.14
Europe	−0.31	1.48	−1.83	−0.52	4.82	1.88
North America	0.32	1.63	−2.11	−1.45	4.43	−2.37
Oceania	−0.57	1.08	−3.42	−2.54	6.14	−2.16
South America	0.35	1.4	−1.8	−0.68	2.89	−2.08

Table depicts mean bias. Bias refers to climatological GSOD minus CHC-CMIP6 versus GSOD minus raw CMIP6 for the local hottest month. CHC-CMIP6 and raw CMIP6 biases are significantly different from each other (Mann-Whitney p-value < 0.01). Global averaged climatological Tmax is similar for GSOD and CHC-CMIP6 (0.08 °C difference), yet CMIP6 T_max_ presents a cool bias (1.59 °C), while less present for T_min_. This indicates that the projected CHC-CMIP6 fields likely contain a cool bias.

### Estimating effect of biases on undercounting of extremes

**Fig. 3:**
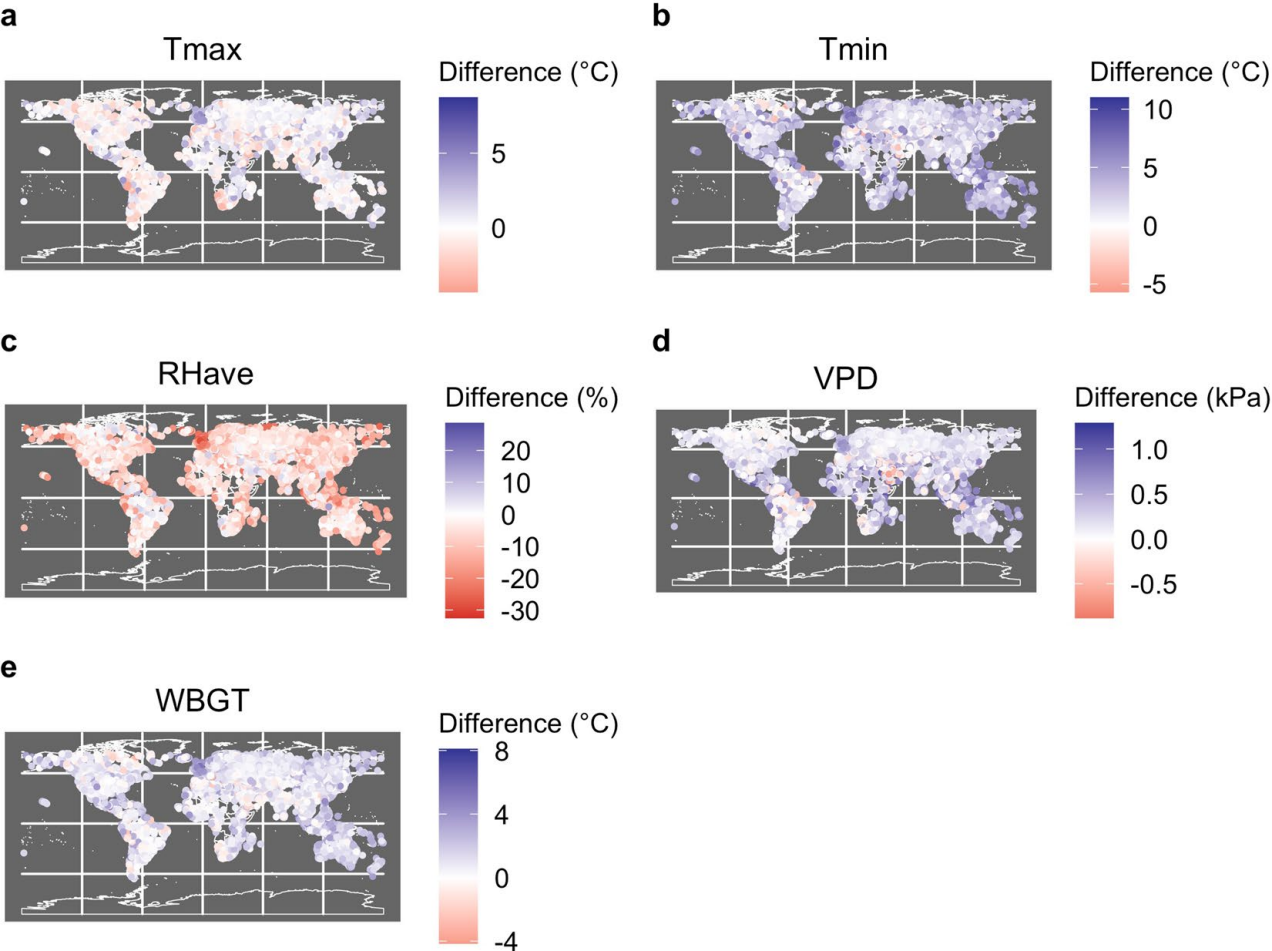
Map of difference between CHC-CMIP6 and GSOD for each variable. Each point depicts the mean difference between CHC-CMIP6 observational fields and GSOD (CHC-CMIP6 minus GSOD) at the location of each GSOD station.

We then tested for potential biases by comparing GSOD with the raw CMIP6 data and CHC-CMIP6 observational data for the 1983–2016 period. First, the multi-model CMIP6 T_min_, T_max_, and RH_ave_ means were calculated from 1983–2016. Then monthly averages were calculated for GSOD and CHC-CMIP6 to compare with the monthly raw CMIP6 data. Next, the differences between GSOD and the two datasets were taken for the three warmest months for each station. The mean biases are reported in Table 3 and shown spatially in Figure 3. To test for statistical significance in difference in biases, a Mann-Whitney test was run comparing the distribution of raw CMIP6 biases and CHC-CMIP6 biases. For cases when the mean bias is smaller for CHC-CMIP6 than for raw-CMIP6, if the Mann-Whitney test p-value is significant, it shows us that the bias in CHC-CMIP6 is statistically significantly lower than the bias in raw CMIP6.

The CHC-CMIP6 biases and raw CMIP6 biases shown in Table 3 are statistically significantly different from each other (Mann-Whitney p-value < 0.05). While GSOD and CHC-CMIP6 T_max_ largely agree, the raw CMIP6 multi-model ensemble average T_max_ is ~1.5 °C cooler than both GSOD and CHC-CMIP6 T_max_. Importantly, as the CHC-CMIP6 projection fields are based on perturbing observational data with CMIP6-derived deltas, this cool bias should be absent from the CHC-CMIP6 projections. For T_min_, the CHC-CMIP6 estimates are about 2 °C warmer than GSOD. This bias may relate to issues with the ERA5 diurnal cycle, which were used to derive the CHC-CMIP6 T_min_ (i.e. CHIRTS_min_) values. The CMIP6 T_min_ values are a little warmer. Hence the raw CMIP6 multi-model ensemble mean is between the GSOD and CHC-CMIP6 mean. However, as the CHC-CMIP6 dataset has been designed to support analysis of temperature-related extremes, the T_max_ values are of greater importance. Finally, the CHC-CMIP6 RH_ave_ is slightly lower than GSOD (~4 percentage points). To determine how large the bias is relative to other RH products used for similar analyses, RH_ave_ climatological fields were retrieved from the gridded, high-resolution observational global climate product, WorldClim^62^. On average, the WorldClim RH_ave_ climatology is ~8 percentage points higher than GSOD. As such, while a bias is present in the CHC-CMIP6 RH_ave_ fields, the fields nonetheless present an improvement compared to other similar products. Note however that the bias will contribute to an undercounting of WBGT_max_ extremes at high temperatures.

Notably, the CMIP6 T_max_ cool bias can lead to a substantial undercounting of VPD and WBGT_max_ extremes. To determine what type of effect such a cool bias might have on T_max_-related projections (i.e. the magnitude of undercounting of extremes by relying solely on CMIP6 data), we perturbed the projected CHC-CMIP6 T_max_ data by subtracting biases of 0 °C, 1.5 °C, and 3 °C. These perturbed estimates, alongside unperturbed estimates, of T_max_ were used to calculate the number of pixels globally experiencing extreme WBGT_max_ and VPD in the 2030_SSP245 and 2050_SSP585 projections, shown in Table 4. The values depicted are for the local hottest day in the first year.

**Table 4:** Estimates of percent of pixels globally experiencing extreme WBGT_max_ and VPD with various T_max_ biases.

Bias	WBGT_max_				VPD			
	2030 SSP245		2050 SSP585		2030 SSP245		2050 SSP585	
	% > 28 C	% > 30 C	% > 28 C	% > 30 C	% > 28 C	% > 30 C	% > 28 C	% > 30 C
0.0	25	7	32	14	29	18	32	20
−1.5	14	2	22	5	26	15	29	17
−3.0	6	0	11	1	23	12	25	14

Percents refer to the number of pixels experiencing an extreme for the local hottest day in the first year.

For the 2050_SSP585 projections, ~14% of the pixels in the CHC-CMIP6 data are over 30 °C WBGT_max_. However, subtracting 1.5 °C from the T_max_ fields used to calculate WBGT_max_ results in only ~5% of the pixels for that same year reaching over 30 °C WBGT_max_. This indicates that a 1.5 °C cool bias in the CMIP6 data leads to a considerable (300%) underestimate of WBGT_max_ extremes. Similarly, the percent of pixels experiencing VPD over 4 kPa decreases from 20% to 17%. While less substantial an increase, the 1.5 °C cool bias also leads to an undercounting of VPD extremes. These VPD biases will have a distinct spatial pattern, preferentially influencing warmer areas due to the exponential relationship between SVP and temperature (VPD). In sum, these results indicate that the CHC-CMIP6 dataset is able to capture projected extremes that would be otherwise missed by relying solely on raw CMIP6 data. Hence, cool bias matters, and has an especially pronounced impact on very extreme WBGT counts, where a 1.5 °C cool bias can dramatically reduce the estimated impact of climate change.

## Usage Notes

When using the CHC-CMIP6 data, please note the following. The CHC-CMIP6 data are available in GeoTiff format, in WGS 1984 projection. Units are degrees Celsius (T_min_, T_max_, WBGT_max_), kPa (VPD), percent (RH_x_, RH_ave_), and mm (precipitation), and all NA values are set to −9999. Finally, please note that, other than the global ocean mask, a water mask was not applied, so users are encouraged to mask out water bodies when using the data.

This is the first iteration of the CHC-CMIP6 dataset. Currently, the observational data are available from 1983–2016. Future versions will include temporally updated data (beyond the current end year in 2016). Furthermore, future versions may include efforts to create a satellite-enhanced RH climatology in an effort to improve the accuracy of the RH fields.

Furthermore, the data descriptor available with the dataset was peer reviewed in 2023 based on the data available on the platform at the time. This data descriptor will be periodically updated for future iterations of the dataset.

### Example Application of Data 1 - Projected Changes in Climatologies and Extremes

Here, we present an example application of the CHC-CMIP6 dataset, examining projected changes in T_max_, RH, VPD, and WBGT_max_ and their associated extremes for some of the highest risk areas for drought and heat stress–the Sahel, southern Africa, and southern Asia.

**Fig. 4:**
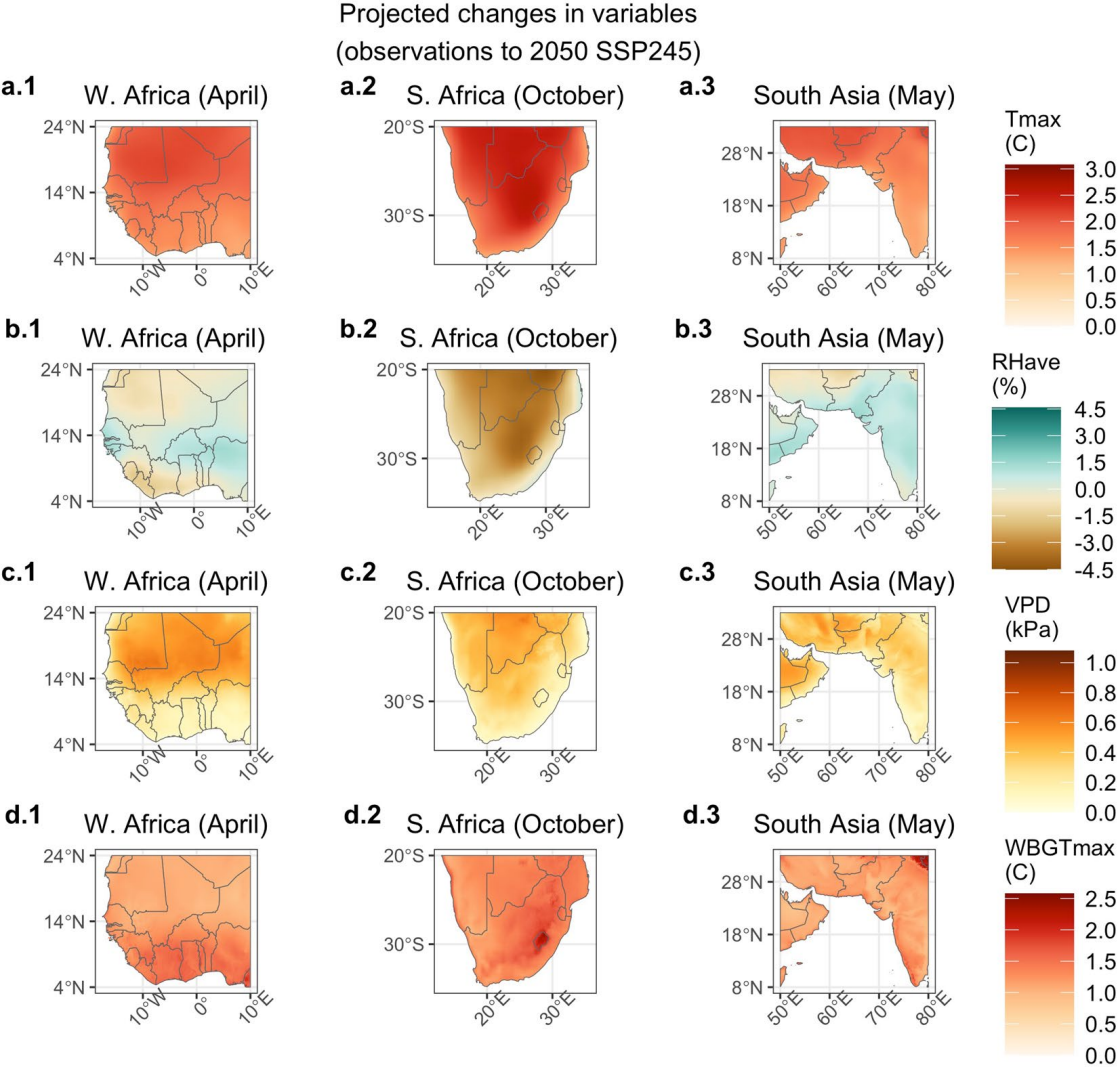
Mean change in T_max_, RH, VPD, and WBGT_max_ from observational period to 2050 SSP245. Deltas are shown for the Sahel in April, southern Africa in October, and southern Asia in May. Months were chosen as the local hottest month and/or shortly before the arrival of the monsoon.

Figure 4 depicts the deltas, or mean changes, in T_max_, RH_ave_, VPD, and WBGT_max_, between the observational and 2050_SSP245 scenario (the ‘most likely’ scenario) for the local hottest month for western Africa (April), southern Africa (October), and southern Asia (May). Each region is projected to experience increases in T_max_. Projected changes in RH_ave_ are more varied: much of South Asia and areas of the Sahel are projected to experience increases in RH_ave_ while southern Africa is projected to experience decreases. The increases in T_max_, coupled with change in RH_ave_, contribute to increases in both VPD and WBGT_max_. However, as expected, the largest projected increases in VPD occur in different locations from the largest projected increases in WBGT_max_.

**Fig. 5:**
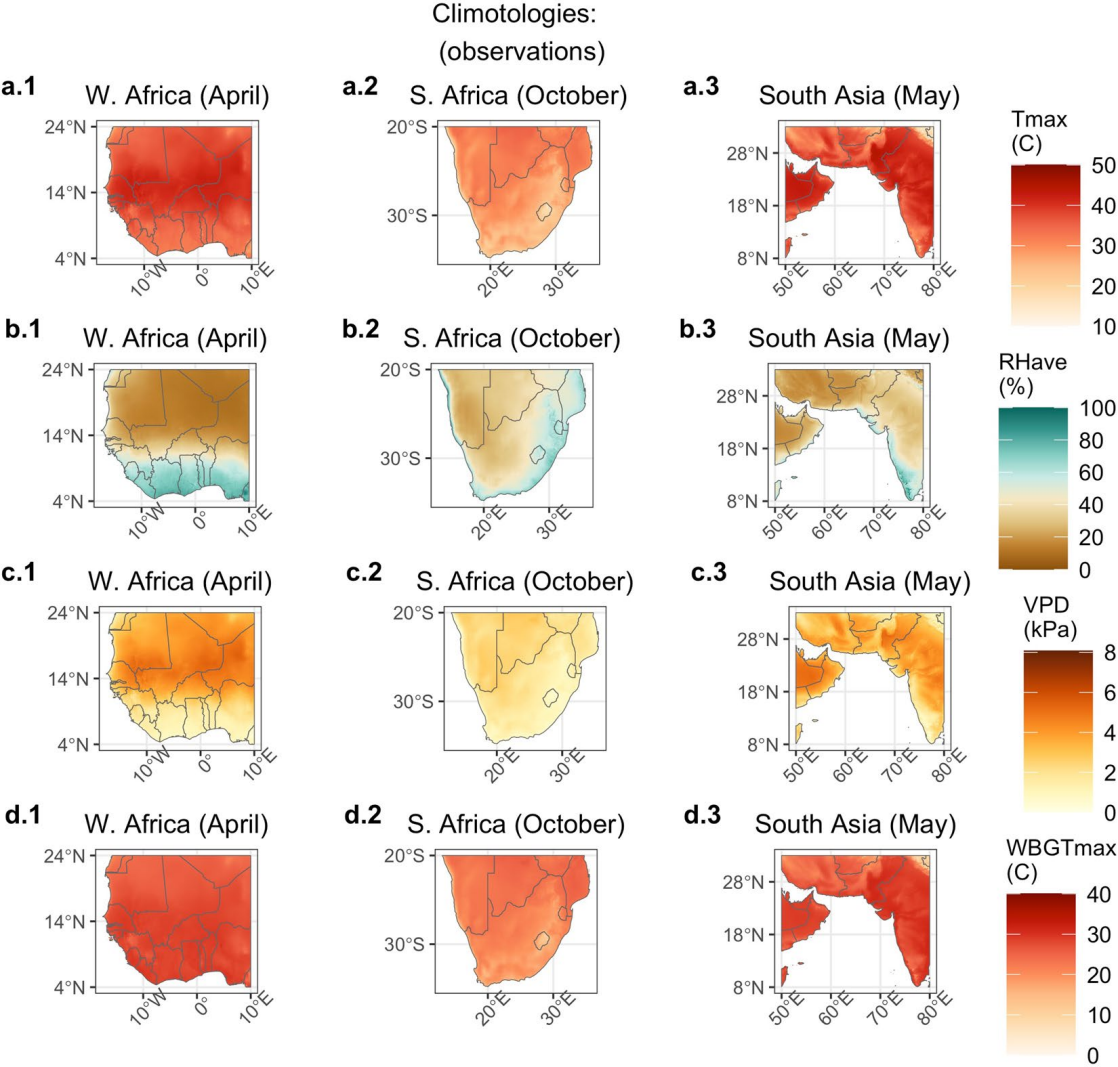
Climatological average T_max_, RH, VPD, and WBGT_max_ for the observational period. Climatological average conditions are shown for the Sahel in April, southern Africa in October, and southern Asia in May.

The largest increases in VPD are seen in the Sahara, inland areas of southern Africa, and in Pakistan and Iran. These areas are projected to experience large increases in T_max,_ accompanied by a decrease or no change in RH_ave_ (except for Pakistan), indicating that increases in T_max_ coupled with reductions in RH_ave_ are driving the change for these locations. These areas have some of the lowest current baseline RH_ave_ climatologies (Fig. 5), suggesting that areas that are already hot and dry can be expected to experience greater increases in VPD, largely due to the exponential relationship between temperature and saturation vapor pressure.

Conversely, the largest increases in WBGT_max_ are seen along the coastal areas south of the Sahel, southeastern South Africa and Lesotho, and southwestern India. Southeastern South Africa and coastal western Africa are projected to experience an increase in T_max_ coupled by a decrease in RH_ave_. Climatological RH_ave_ values are already high for these places, so despite a projected decrease in RH_ave_, the increase in T_max_ drives an overall increase in WBGT_max_. This indicates that due to the non-linear relationship between temperature, relative humidity, and WBGT_max_, these already hot-humid areas are projected to experience some of the greatest increases in WBGT_max_. For India, both T_max_ and RH_ave_ are projected to increase, together driving the projected increase in WBGT_max_. Note that while much of India experiences a large increase in T_max_ and an increase in RH_ave_, the WBGT_max_ increase is somewhat muted—this may be because our estimation of WBGT_max_ becomes asymptotic at very high levels of WBGT_max_ (see Fig. 4 in^7^ and Fig. 1 in^50^). Also note that though our parameterization of WBGT_max_ differs, previous work^7^ found some locations in southern and southeastern Asia are saturating, whereby the vast majority of days in 2016 exceed WBGT_max_ of 30 °C and thus these areas cannot face an annual increase going forward.

**Fig. 6:**
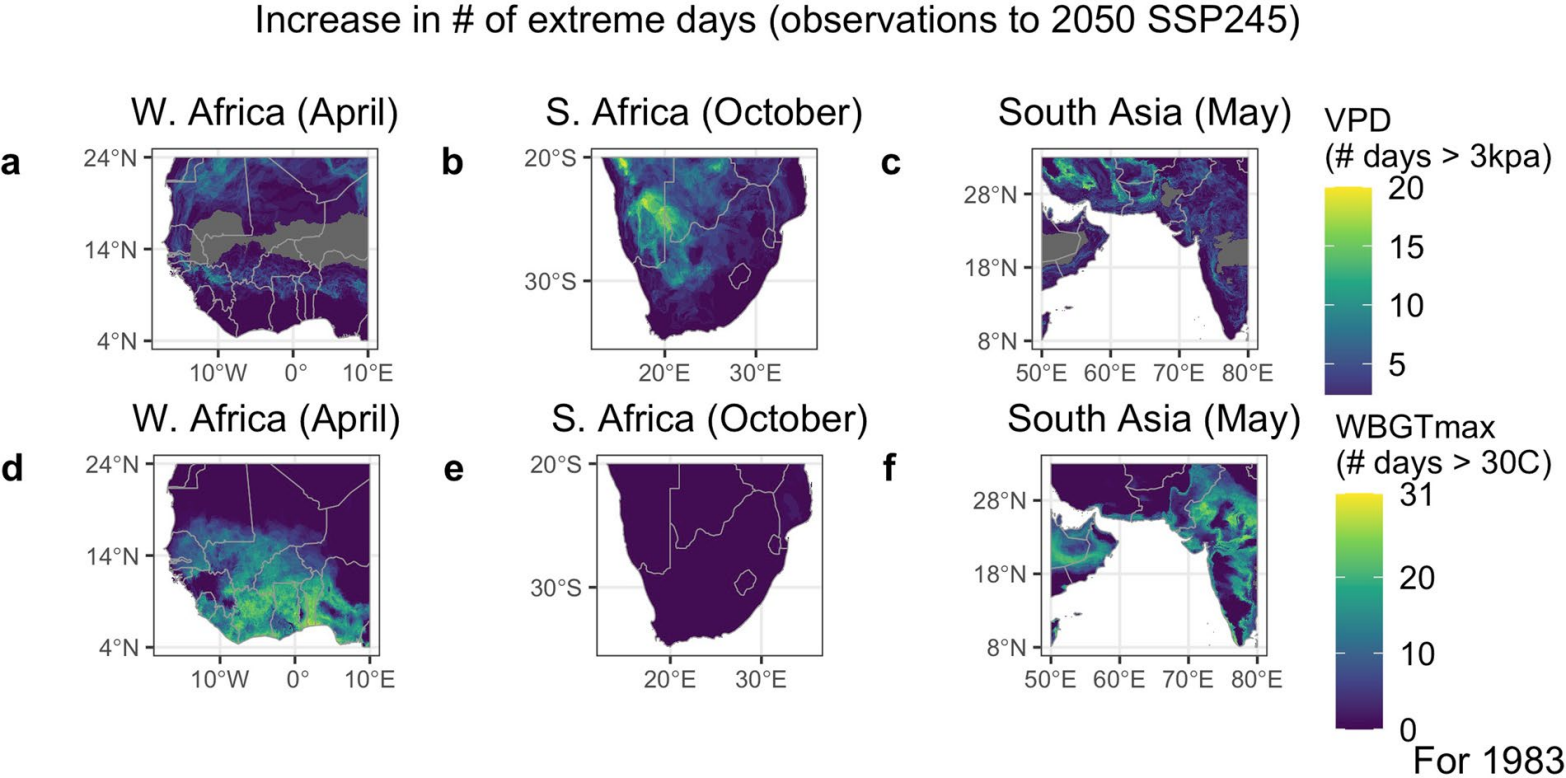
Change in the number of ‘extreme’ days between the first year (1983) of the observational and 2050 SSP245 scenarios. The extremes are defined as surpassing the 3 kPa threshold for VPD and the 30 °C threshold for WBGT_max_. The increase in the number of extreme days is depicted for the local hottest month for the Sahel in April (right column), southern Africa in October (middle column), and southern Asia in May (right column). Note, locations that already experience extremes for all days of the month are grayed out (e.g. across the Sahara for VPD).

The projected increase in the number of extreme events for VPD (3 kPa threshold) and WBGT_max_ (30 °C threshold) from the observational period to 2050_SSP245 for the local hottest month are shown in Fig. 6. Regions that already experience extremes for every day in the observational climatology are grayed out. The patterns largely correspond to the patterns shown in Figs. 4, 5—the hot-humid areas (the Sahel, India) are projected to experience a substantial increase in frequency of WBGT_max_ extreme days. Conversely, the hot-dry areas of inland southern Africa are projected to experience a large increase in the number of days with high VPD levels.

Daily extremes for WBGT_max_ can have direct, acute physiological implications—each day above the corresponding threshold can increase the risk of heat stress for local populations^63^. However, for VPD, it is the cumulative effect of numerous days over extreme values over weeks to months that results in lowered crop yield or pasture productivity.

### Example Application of Data 2 - Extreme July T_max_ Counts in India-Pakistan

**Fig. 7:**
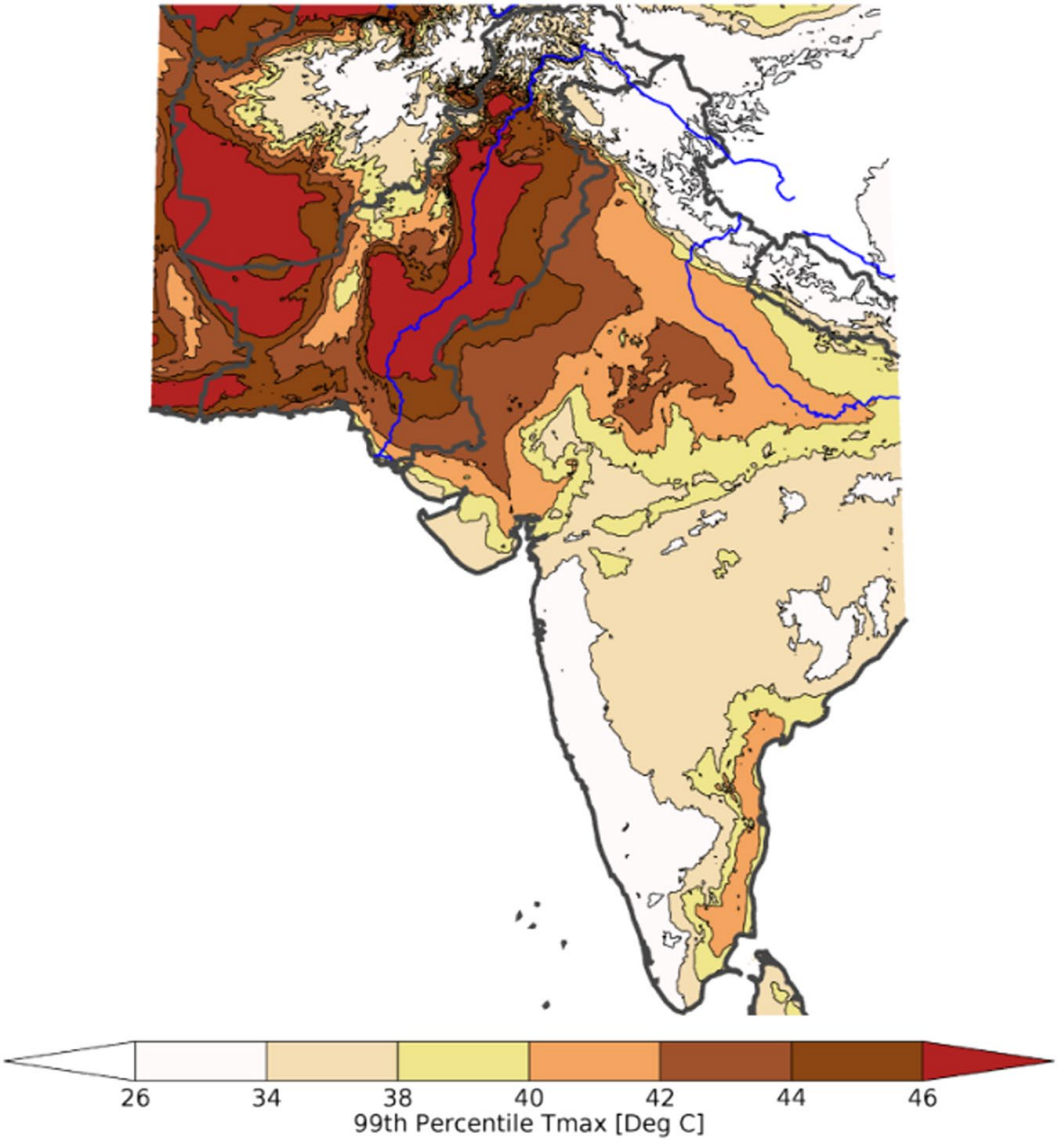
Observed 99th percentile July Tmax values. Based on observed CHIRTS daily Tmax data.

Here we also include a brief examination of 99th percentile temperature changes over the India-Pakistan region. The 99th percentile values are based on time series 34 × 31 days (1,054). Nevertheless, there will still be substantial uncertainty surrounding the estimated extreme value quantile values^60^, as one percent of the observations only corresponds to ~10 values. On the other hand, this uncertainty should not preclude the identification of high risk areas, as these fields can still be used to map risk. For example, Fig. 7 displays the estimated, observed 99th percentile T_max_ values for a very warm area in July in Pakistan and India. While the individual 99th percentile values will be associated with substantial uncertainty, the spatial distribution of extreme temperatures are clear. The extreme temperatures range from less than 26 °C to more than 46 °C. Many of the warmest areas, such as the low-lying, densely populated Indus River basin in Pakistan, are already associated with extreme warm extreme T_max_ values.

**Fig. 8:**
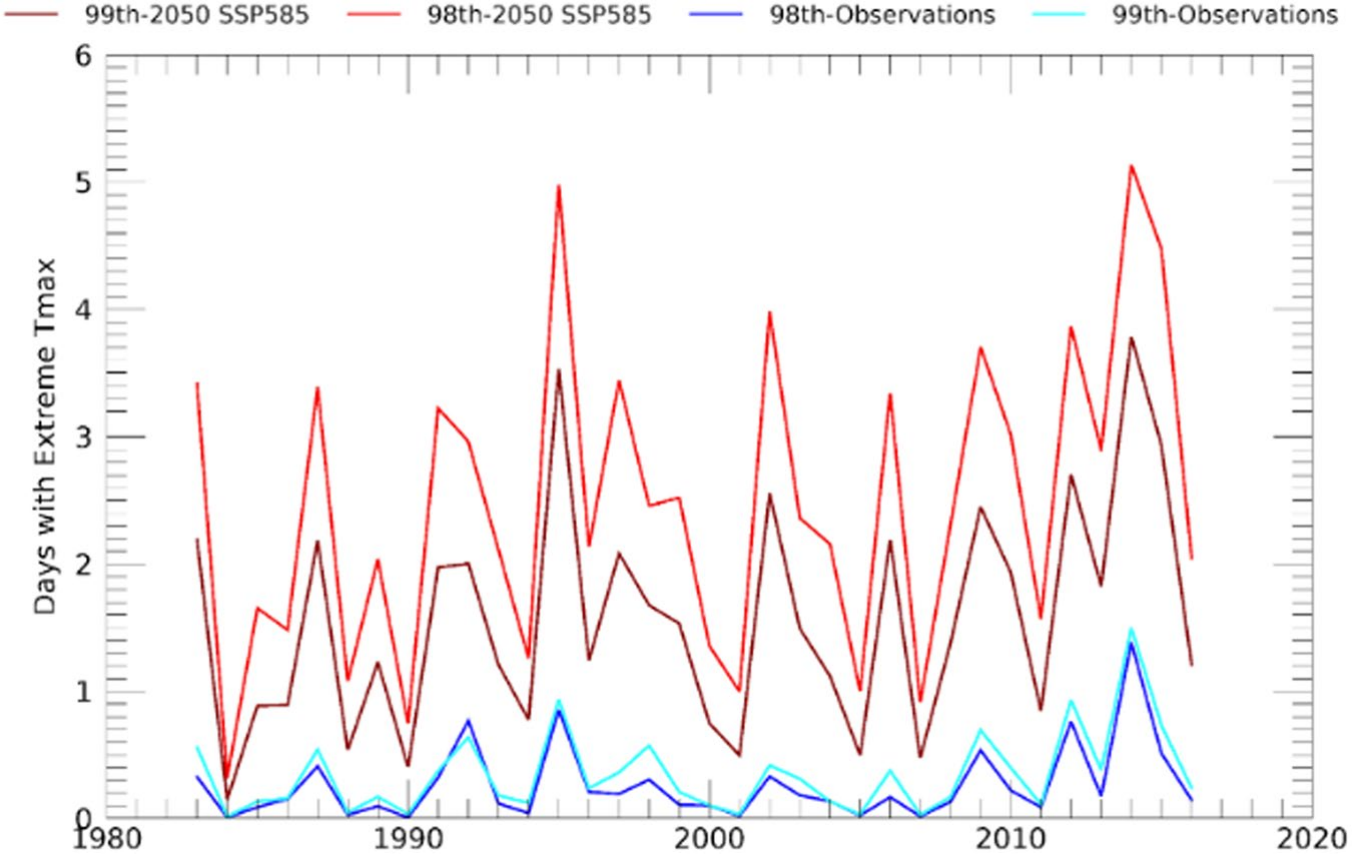
Average number of days in July exceeding the 98th and 99th percentile values for the India-Pakistan region shown in Fig. 7.

The monthly extreme counts data can be used to examine changes in the frequency of very warm (>99th percentile) days. For the region displayed in Fig. 7 (50–85°E, 8–37°N), Fig. 8 displays the mean number of days exceeding the 99th percentiles in the observations and the SSP585 2050 warming scenario. While not part of the pre-computed data set, for illustrative purposes, we also plot on this figure similar counts based on the 98th percentile quantile values: both the 98th and 99th quantile-based counts are very similar. By construction, between 1983 and 2010, the mean number of observed days warmer than the 98th and 99th quantile values will be low. However, when we contrast the observed mean 2011–2016 and 1983–2011 extreme counts, we very similar changes in the 98th and 99th percentile time-series - with a 240% and 230% increase in the frequency of very warm days.

Looking at the 2050 SSP585 projections shown in Fig. 8, the 98th and 99th percentile counts both exhibit further dramatic increases. According to these projections, comparing the 1983–2016 July time-series in Fig. 8, the number of days exceeding the 98th and 99th percentile thresholds will increase by five-fold or more over the observed 1983–2016 frequencies. Thus, while caution should be taken when considering counts of very extreme values, this data set can provide a useful foundation for assessing risk, given the strong performance of the CHIRTS data, and the fact that both the observed and projected changes are very large.

## Supplementary information

Supplementary Information

## Data Availability

The equations used to calculate RH, VPD, and WBGT_max_ are available in R on GitHub (https://github.com/emilylynnwilliams/CHC-CMIP6_SourceCode). The CHC-CMIP6 dataset was processed using code written in the Interactive Data Language and Python.
