# Supplementary material for: High resolution climate change observations and projections for the evaluation of heat-related extremes
Source: Sci Data. 2024 Mar 1;11:261. doi: 10.1038/s41597-024-03074-w (PMC11422495; doi:10.1038/s41597-024-03074-w)
Supplement: Supplementary file 1 — Supplementary Information [file 41597_2024_3074_MOESM1_ESM.pdf]

High resolution climate change observations and projections for the evaluation of heat-related extremes

Supplementary Information

Emily Williams[1,2], Chris Funk[1], Pete Peterson[1], Cascade Tuholske[3,4]

[1] Climate Hazards Center, University of California, Santa Barbara, 93106, USA
[2] Sierra Nevada Research Institute, University of California, Merced, CA 95343, USA
[3] Department of Earth Sciences, Montana State University, 59717, USA
[4] Geospatial Core Facility, Montana State University, 59717, USA

Correspondence to: Emily L. Williams (emilywilliams@ucmerced.edu) & Chris Funk (chris.funk@geog.ucsb.edu)

Contents:

- Figure S1 (p.2): Stations available for the United States and across Africa.
- Figure S2 (p.4): $WBGT_{max}$ and HI_{max} values for hot-dry conditions.
- Figure S3 (p.5): $WBGT_{max}$ and HI_{max} values for hot-wet conditions.
- Text S1 (p.2): $WBGT_{max}$ calculation notes.
- Text S2 (p.3): $WBGT_{max}$ and HI_{max} uncertainty.

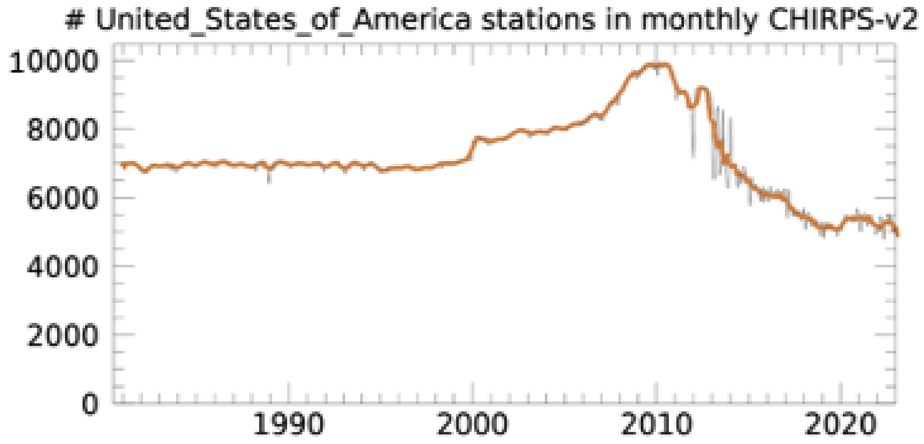

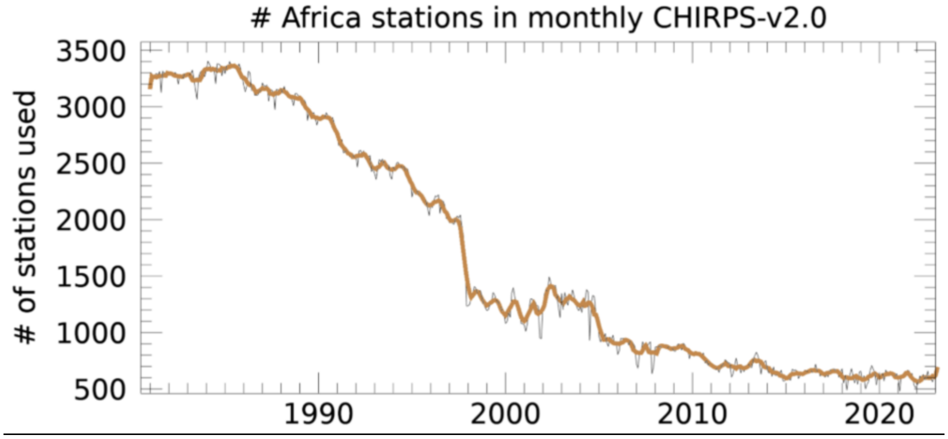

Figure S1. Stations available for use in CHIRPS-v2 for the United States (top panel) and across Africa (bottom panel). The number of stations available for Africa has declined dramatically since the 1990s.

Text S1: $WBGT_{max}$

The observational daily maximum wet bulb globe temperature ($WBGT_{max}$) data in the CHC-CMIP6 differs from Tuholske *et al.* (2021) for two reasons. First, we updated our parameterization of relative humidity (RH) to only use ERA-5 dew point temperature T_d following Daly et al. 2015. Second, here we estimate RH at the hour at which daily maximum temperature occurred (T_{max}), e.g. RH_x, to better represent relative humidity at the time peak air temperatures tend to occur (Davis *et al.* 2016). In Tuholske et al. (2021), T_{max} and daily minimum relative humidity (RH_{min}) were used to construct $WBGT_{max}$ following the same process discussed in Section 2.1.2. However, in Tuholske *et al.* 2021, RH_{min} was calculated by first estimating daily average actual vapor pressure (AVP) with with down-scaled ERA5 dew-point pressure (T_d) and surface pressure (*p*, kg/kg) from MERRA-2 (Tuholske et al. 2021; Bolton et al 1980). To derive RH_{min}, average AVP was then divided by saturation vapor pressure (SVP), which was estimated with CHIRTS-daily T_{max}. The new parametrization of RH_x better aligns T_{max} and RH in the diurnal cycle (Davis *et al.* 2016) to generate $WBGT_{max}$ and simplifies the process by using only T_d from ERA-5, not requiring inputs from two different reanalysis products.

Text S2: HI_{max} and $WBGT_{max}$ Uncertainty

To demonstrate possible uncertainty from combinations for daily maximum heat index (HI_{max}) and $WBGT_{max}$ from RH and T, we plot all possible values for extreme hot-dry (35°C >= T <= 50°C and 0% >= RH <= 25%) and hot-humid conditions (25°C >= T <= 35°C and 25% >= RH <= 75%) in Figures S2 and 3, respectively.

Errors may arise when estimating HI from uncertainty in estimating both RH and T due to the nonlinear exponential scaling of both RH and T when calculating HI with the US National Weather Services Heat Index equation (NOAA 2022). For example, under hot-dry conditions, at T=35°C, the difference between RH of 12% and RH of 25% only leads to a 2°C change in HI (Figure S2B). But when T = 40°C, increasing RH from 12% to 25% leads to an increase in HI of 4°C (Figure S2B). Similarly, with hot-humid conditions of 35°C, an increase of RH from 50% to 65%, will lead to a 9°C increase in HI (Figure S3B). For both hot-dry and hot-humid conditions, even 2-3% error in RH can lead to a ±2°C error in HI estimation at higher T.

Errors due to RH are less-pronounced when estimating $WBGT_{max}$ because of the asymptotic nature of the quadratic function we employ (see Figure 1 in Bernard and Iheanacho 2015). Using the same example as HI, when T=35°C, the difference between RH of 12% and RH of 25% only leads to a 1°C change in WBGT (Figure S2A). With hot-humid conditions, at T = 35°C, an increase of RH from 50% to 60%, will lead to a 2°C increase in WBGT (Figure S2B). Unlike HI, for both hot-dry and hot-humid conditions, a 2-3% error in RH will lead to a <= ±1°C error in WBGT estimation at higher T. We note that due to the quadratic function we employ to transform HI to WBGT (Bernard and Iheanacho 2015), with very hot-humid conditions (T >=40°C and RH >60%), WBGT can actually be lower than WBGT at lower RH ranges (T >>=40°C and RH <50%).

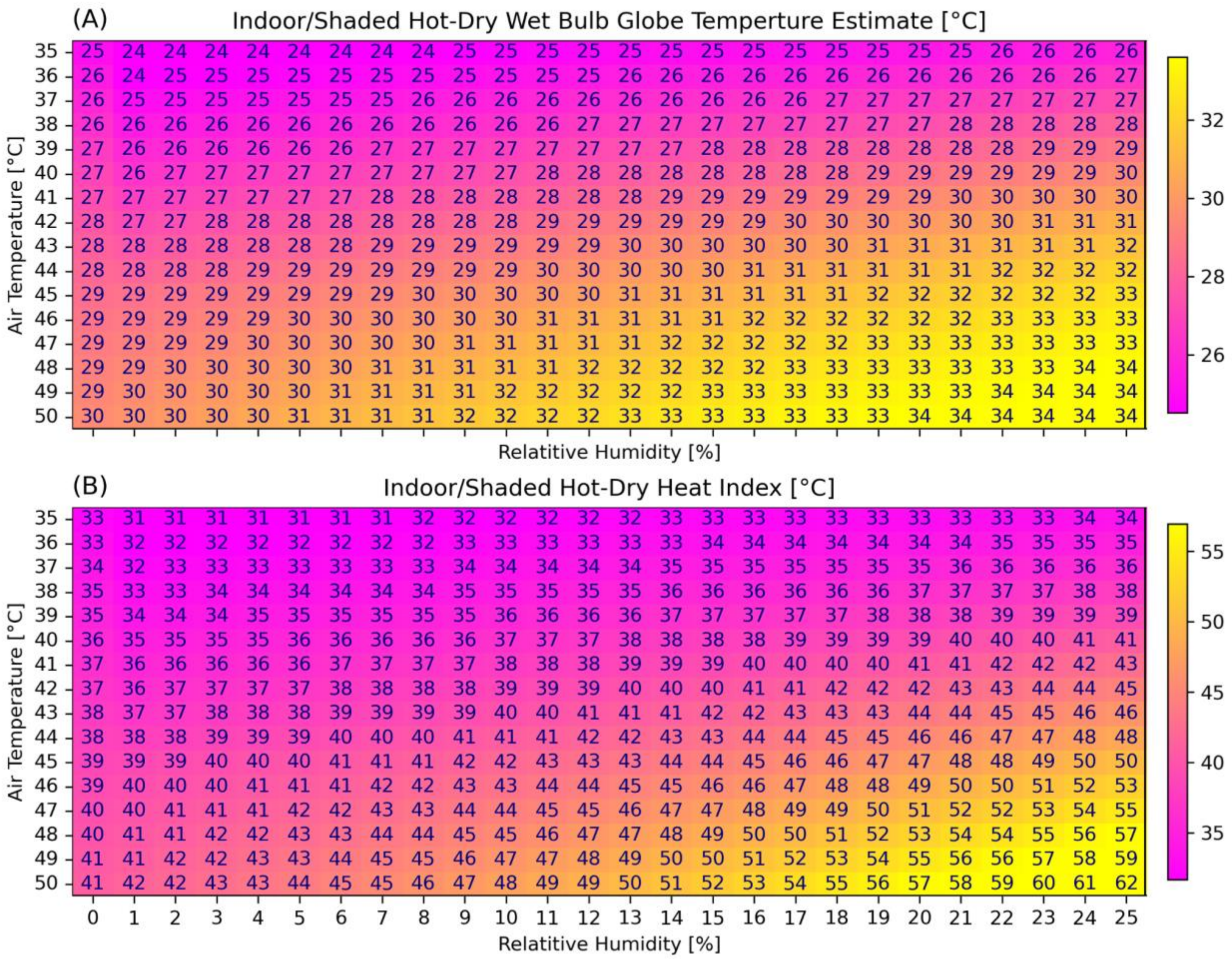

Figure S2. Range of possible shaded/indoor (A) wet bulb globe temperature and (B) heat index values for extreme hot-dry conditions (35°C >= T <= 50°C and 0% >= RH <= 25%).

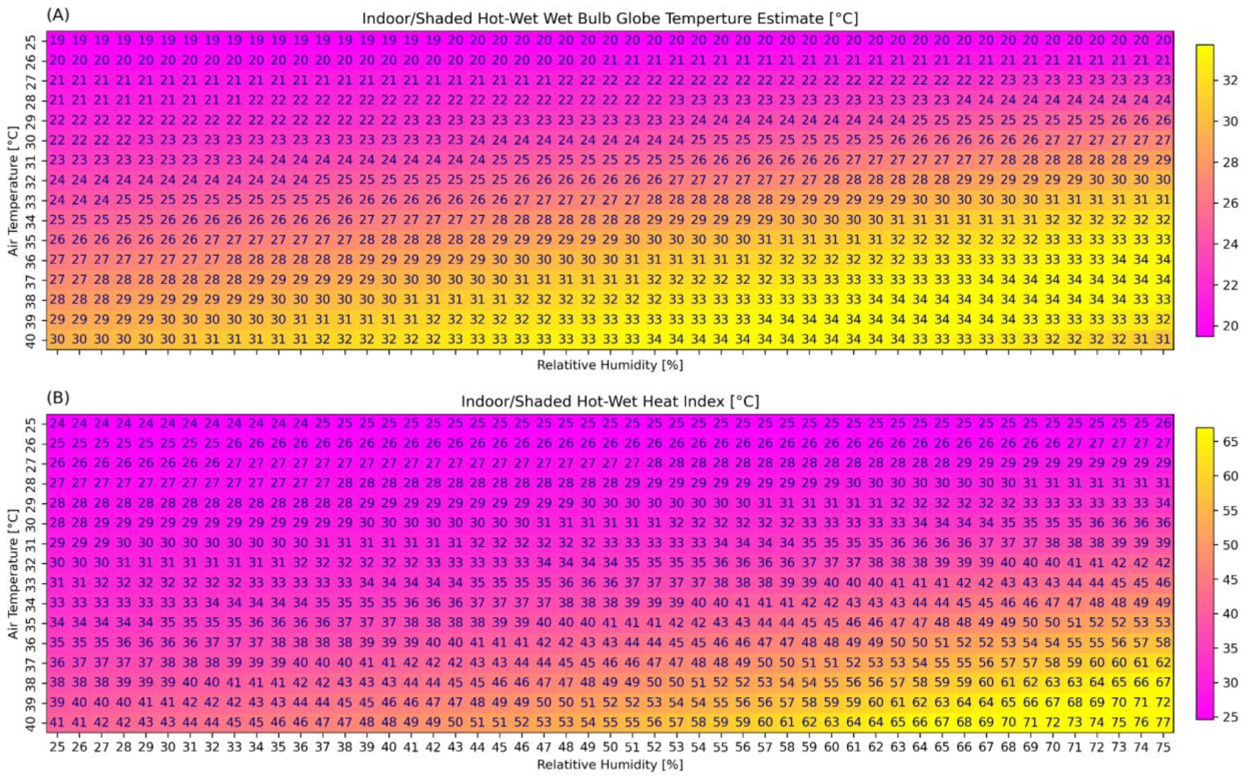

Figure S3. Range of possible shaded/indoor (A) wet bulb globe temperature and (B) heat index values for extreme hot-wet conditions (25°C >= T <= 35°C and 25% >= RH <= 75%).

References

Bernard, T. E. and Iheanacho, I. Heat index and adjusted temperature as surrogates for wet bulb globe temperature to screen for occupational heat stress. *JOEH*. **12,** 323-333. (2015).

Bolton, D. (1980). The computation of equivalent potential temperature. Monthly weather review, 108(7), 1046-1053.

Davis, R. E., McGregor, G. R., & Enfield, K. B. Humidity: A review and primer on atmospheric moisture and human health. Environmental research. **144,** 106-116. (2016).

NOAA. The heat index equation. https://www.wpc.ncep.noaa.gov/html/heatindex_equation.shtml (2022)
Tuholske, C., et al. Global urban population exposure to extreme heat. *PNAS*. **118,** 1-9. (2021).
